# Genetic, vascular and amyloid components of cerebral blood flow in a preclinical population

**DOI:** 10.1177/0271678X231178993

**Published:** 2023-05-26

**Authors:** Beatriz E Padrela, Luigi Lorenzini, Lyduine E Collij, David Vállez García, Emma Coomans, Silvia Ingala, Jori Tomassen, Quinten Deckers, Mahnaz Shekari, Eco JC de Geus, Elsmarieke van de Giessen, Mara ten Kate, Pieter Jelle Visser, Frederik Barkhof, Jan Petr, Anouk den Braber, Henk JMM Mutsaerts

**Affiliations:** 1 Department of Radiology and Nuclear Medicine, Amsterdam Neuroscience, Amsterdam University Medical Center, Location VUmc, Amsterdam, the Netherlands; 2 Amsterdam Neuroscience, Brain Imaging, Amsterdam, the Netherlands; 3 Alzheimer Center Amsterdam, Neurology, Vrije Universiteit Amsterdam, Amsterdam UMC location VUmc, Amsterdam, the Netherlands; 4 Amsterdam Neuroscience, Neurodegeneration, Amsterdam, the Netherlands; 5 BBRC: Barcelonaβeta Brain Research Center (BBRC), Pasqual Maragall Foundation, Barcelona, Spain; 6 Pompeu Fabra University, Barcelona, Spain; 7 IMIM (Hospital del Mar Medical Research Institute), Barcelona, Spain; 8 Department of Biological Psychology, Vrije Universiteit Amsterdam, Amsterdam, the Netherlands; 9 Alzheimer Center Limburg, School for Mental Health and Neuroscience, Maastricht University, Maastricht, the Netherlands; 10 Queen Square Institute of Neurology and Centre for Medical Image Computing (CMIC), University College London, London, UK; 11 Helmholtz-Zentrum Dresden-Rossendorf, Institute of Radiopharmaceutical Cancer Research 28414 , Dresden, Germany

**Keywords:** Arterial spin labeling (ASL), cerebral blood flow (CBF), twin analysis, white matter hyperintensities, Alzheimer’s disease

## Abstract

Aging-related cognitive decline can be accelerated by a combination of genetic factors, cardiovascular and cerebrovascular dysfunction, and amyloid-β burden. Whereas cerebral blood flow (CBF) has been studied as a potential early biomarker of cognitive decline, its normal variability in healthy elderly is less known. In this study, we investigated the contribution of genetic, vascular, and amyloid-β components of CBF in a cognitively unimpaired (CU) population of monozygotic older twins. We included 134 participants who underwent arterial spin labeling (ASL) MRI and [^18^F]flutemetamol amyloid-PET imaging at baseline and after a four-year follow-up. Generalized estimating equations were used to investigate the associations of amyloid burden and white matter hyperintensities with CBF. We showed that, in CU individuals, CBF: 1) has a genetic component, as within-pair similarities in CBF values were moderate and significant (ICC > 0.40); 2) is negatively associated with cerebrovascular damage; and 3) is positively associated with the interaction between cardiovascular risk scores and early amyloid-β burden, which may reflect a vascular compensatory response of CBF to early amyloid-β accumulation. These findings encourage future studies to account for multiple interactions with CBF in disease trajectory analyses.

## Introduction

Age-related cognitive decline is a multifactorial process influenced by a combination of genetic determinants,^1^ cerebrovascular dysfunction,^2^ and amyloid-β (Aβ) accumulation.^3^ While up to 80% of the variability in Alzheimer’s disease (AD) is explained by genetic factors,^1^ the mechanisms leading from genetic vulnerability to vascular dysfunction and Aβ-plaque formation are still poorly understood. These disease factors seem to be correlated with cerebral blood flow (CBF) in the late symptomatic stages of AD and cerebral small vessel disease (SVD), arguably the most relevant pathological conditions related to cognitive decline. CBF has the potential to be an early biomarker of cognitive decline as it is implicated in the supply (reflecting vascular robustness) and demand (neuronal metabolism) of blood to the brain. Arterial spin labeling (ASL) is a non-invasive MRI technique that provides *in-vivo* quantitative measurements of CBF.^4,5^ To date, it has not been fully clarified to what extent these disease factors explain underlying components of CBF, especially in the pre- or pauci-symptomatic stages of age-related cognitive dysfunction.

The (patho-)physiology of AD and SVD seems to be interconnected,^6–8^ and it has been suggested that vascular dysfunction is a prominent feature in the AD cascade.^9,10^ The risk of SVD can be quantified by the clinical Framingham risk score (FRS), while its extent can be assessed by the presence of white matter hyperintensities (WMH) on MRI. An inverse association between CBF and WMH has been reported in symptomatic stages of SVD^11^ and AD,^12^ but limited studies are available regarding this association in the asymptomatic stages.

Similarly, Aβ pathology has been shown to be related to a decrease in global CBF in symptomatic stages (measured with ASL),^13,14^ but inconsistent findings have been reported for earlier stages.^15^ Importantly, in preclinical populations, it has been proposed that the deposition of Aβ in AD follows a consistent spatial-temporal sequence, starting from the precuneus, basal-frontal areas, and the cingulate cortex (“early accumulation region”).^16,17^ Therefore, the relationship between Aβ and CBF might follow such spatial patterns. The regional correspondence between Aβ deposition and CBF changes remains unclear.

In the same line, genetic determinants have been related to Aβ deposition, WMH burden,^18^ and global CBF,^19^ but literature reports regarding the genetic contribution to *regional* CBF are limited. A unique insight can be obtained by studying genetically identical twin pairs. To this aim, we investigated (1) the genetic component of CBF by evaluating twin-pair similarities with both global CBF measures and CBF patterns in pre-defined anatomical territories, and (2) the relationship between CBF and WMH and Aβ burden, with and without the interaction effect of FRS, in a cognitively unimpaired (CU) population using both cross-sectional and longitudinal data.

## Material and methods

### Study participants

Data were drawn from the prospective Amsterdam substudy of the European Medical Information Framework for Alzheimer's Disease (EMIF-AD) PreclinAD Twin60++ cohort.^20^ Detailed inclusion and exclusion criteria have been previously described.^20^ Briefly, the main inclusion criteria were aged 60 years and older and a Clinical Dementia Rating score of 0. Participants were recruited between December 2014 and August 2016 from the Netherlands Twin Registry.^21^ The Medical Ethics Review Committee of the VU University Medical Center performed approval of the study in Amsterdam. Research was performed according to the principles of the Declaration of Helsinki and in accordance with the Medical Research Involving Human Subjects Act and codes on ‘good use’ of clinical data and biological samples as developed by the Dutch Federation of Medical Scientific Societies. All participants gave written informed consent. For the follow-up acquisition 4 years after baseline, participants were scanned again with an identical scanner, scanner software, and scan protocol. Follow-up scanning was performed as part of the Amyloid Imaging to Prevent Alzheimer’s disease Prognostic and Natural History (AMYPAD-PNHS) study.^22^ Out of the total 204 participants, we included all subjects that had PET, MRI, all parameters to calculate the cardiovascular risk scores, and a good-quality ASL scan (n = 134 for baseline and n = 88 for follow-up, Supplementary figure 1).

### Framingham risk score

The cardiovascular risk profile for each participant was defined by the Framingham risk scores (FRS) index.^23^ The FRS index estimates the 10-year cardiovascular risk of an individual, using information on age, sex, systolic blood pressure, use of anti-hypertensive medication, diabetes, total- and high-density lipoprotein cholesterol, and smoking. In the absence of blood biomarkers, self-reported hypercholesterolemia was used to score cholesterol-related information and re-coded as described previously.^24^ While the FRS is clinically used to predict the 10-year risk for cardiovascular events, here we use the baseline FRS as a composite proxy score for the current cardiovascular health.^25,26^ To separately investigate non-cardiovascular age effects, we excluded age from the FRS calculation.

### PET acquisition and processing

[^18^F]Flutemetamol amyloid-PET scans were used to image cortical Aβ burden. PET scans were performed using a Philips Ingenuity Time-of-Flight PET–MRI scanner. All participants were scanned with the dual-time window acquisition protocol,^27^ i.e., from 0 to 30 min and then again from 90 to 110 min after intravenous injection of 185 MBq (±10%) [^18^F]flutemetamol.^20^ All scans were checked for movement, and the late-acquisition frames were summed to obtain a static image (90–110 min). All scans were quantified using the Centiloid (CL) method.^28^ This scale is anchored on [^11^C]PiB standardized uptake value ratio data and constructed such that CL = 0 represents the mean level of amyloid-PET tracer uptake in young controls, while CL = 100 reflects the average signal observed in typical mild-to-moderate AD dementia patients. Centiloid values were obtained from both global (standard GAAIN target region), and four early Aβ accumulation regions-of-interest (ROIs) obtained from the LEAP atlas:^22,29^ precuneus, basal-orbital frontal gyrus, superior frontal gyrus, and lingual gyrus (Supplementary figure 2).

Images were also visually rated (VR) according to the GE Healthcare reader guidelines.^30^ Aβ status was rated in a consensus read between three trained readers for baseline and two trained readers for follow-up. In case of discordance, a consensus read was met. Positive Aβ status (VR+) was defined as unilateral binding in ≥1 of the five regions of interest, including the frontal cortex, precuneus/posterior cingulate (PC/PCC), lateral-parietal, lateral temporal, and striatum — whereas negative Aβ status (VR−) was assigned in case of predominantly white matter uptake.

### MRI acquisition and processing

MRI scans were acquired using a 3T Ingenuity Time-of-Flight PET/MRI scanner (Philips Healthcare, Best, The Netherlands) with an 8-channel head coil. The scan protocol included a 3D T1-weighted scan (1.00 × 1.00 × 1.00 mm^3^ voxels), a 3D FLAIR scan (1.12 × 1.12 × 1.12 mm^3^ voxels),^18^ and a 2D EPI pseudo-continuous arterial spin labeling (PCASL) scan, acquired with post-labeling delay (PLD) = 2025 ms for the first slice, slice timing = 38.3 ms (60 slices), labeling duration = 1650 ms, two background suppression pulses = 1710 and 3142 ms, TR = 4.56 s, TE = 13.9 ms, control-label pairs = 30). WMH volume was obtained from the 3D FLAIR scans using the Bayesian Model Selection (BaMoS) method.^31^ Additionally, microbleeds were visually assessed on susceptibility-weighted imaging (SWI) scans (0.8 × 0.8 × 1.20 mm^3^), defined as rounded hypointense homogeneous foci of up to 10 mm in the brain parenchyma. Lacunes were defined as deep lesions from 3 to 15 mm with CSF-like signal, visually assessed on T1-weighted and FLAIR image.^20^

ASL image processing was performed using ExploreASL version 1.8.0.^32^ Briefly, T1w images were segmented into gray matter (GM), white matter (WM), and cerebrospinal fluid using the Computational Anatomy Toolbox 12.^33^ ASL images were motion corrected, outliers excluded and rigid-body registered with T1w. CBF quantification was performed using the single-compartment model^34^ after M0 division. Mean regional CBF was obtained from several ROIs, and all CBF values are for the GM (using a pGM > 0.7 threshold). Additionally, the WM CBF was obtained from an eroded WM mask^32^)

We used the following ROIs for different analyses. For the genetic and vascular components of CBF we used the total GM and 3 ROIs covering the vascular territories supplied by the anterior (ACA), middle (MCA), and posterior cerebral artery (PCA).^35^ For the genetics of CBF patterns, we included ROIs from the Hammers’ atlas,^36^ in order to obtain patterns of perfusion based on smaller ROIs. For the correlation with amyloid, we used the division of the vascular territories that supply the amyloid-PET ROIs, previously separated into proximal, intermediate, and distal ROIs based on arterial transit time.^35^ Specifically, were used: 1) ACA distal for the precuneus; 2) MCA intermediate for the orbital-frontal gyrus; 3) ACA intermediate for the superior frontal gyrus; and 4) PCA intermediate for the lingual gyrus (Supplementary figure 2).

### Statistical analysis

All analyses were performed for baseline CBF and delta CBF (follow-up minus baseline, divided by time). All statistical analyses were performed in R Statistical Software (v3.3.1; R Core Team 2021), with p < 0.05 considered as statistically significant, and, in order to remove outliers bias, we tested the same analyses for the same data without values higher or lower than mean The first analysis (Genetic component of CBF) was applied only to the complete twin pairs of the cohort, whereas the second analysis (Cardiovascular, cerebrovascular, and amyloid proteinopathy components of CBF) included all of the participants.

#### Genetic component of CBF

To study the genetic component of CBF, pairwise CBF similarities were estimated using a one-way single-measure intraclass correlations (ICC) analysis for each ROI (global, ACA, MCA, and PCA, respectively). Normalized CBF values were also tested (vascular territories CBF divided by total GM CBF). This analysis provides an estimate of the upper limit of the genetic contribution to a trait, as the monozygotic twins are genetically identical. ICC’s were estimated for the 64 twin pairs at baseline and 39 twin pairs at follow-up. To create a comparative reference distribution, we created sets of random (i.e, non-Twin) pairs, and repeated the ICC analysis for 20 different sets of these random pairs.^37^

To assess whether twin pairs show a more similar CBF spatial distribution than non-twin pairs, we extracted the CBF values of each Hammers atlas’ ROI (larger than 1 mL, n = 52 regions), for every participant. Then, we correlated each participant’s CBF spatial distribution to that of every other participant using a Spearman correlation model. A paired t-test was performed to assess whether the average correlation coefficient obtained for twin pairs was significantly higher than the average correlation coefficient obtained for non-twin participant pairings. This yields an estimate of whether twin pairs show a more similar CBF spatial distribution than non-twin pairs.

#### Cardiovascular, cerebrovascular, and amyloid proteinopathy components of CBF

To investigate the association of cardiovascular, cerebrovascular, and Aβ with CBF, analyses were adjusted for age, sex, and twin dependency using the General Estimations Equations (GEE) model, with the unstructured correlation as the working correlation matrix and the Wald test to calculate p-values.

First, we examined the CBF associations with FRS (cardiovascular), WMH volume (cerebrovascular), and their interaction. Second, we examined the CBF differences between microbleed count groups (0, 1–2, or more than 2)^38^ using the Kruskal-Wallis test and between lacune count groups (0 or 1) with Mann-Whitney-Wilcoxon Test. The choice for non-parametric testing was made to account for the group size differences. Third, we examined the CBF associations with Aβ as well as its interactions with FRS and WMH. Because of the specific regional vulnerability of Aβ,^16,17^ we compared four early Aβ ROIs with the CBF of their spatially overlapping vascular territories, as explained in section 2.4. As a post-hoc analysis, we longitudinally divided the Aβ groups into Stable Aβ−, Converters to Aβ+, and Stable Aβ+, according to the VR at baseline and follow-up.

#### Influence of APOE4

As a post-hoc analysis, we investigated the influence of the APOE genotype on the association of CBF with amyloid burden or WMH volume, by repeating the GEE analyses with APOE4 carrier status (0 or 1), adjusted for age, sex, and twin dependency.

## Results

### Cohort characteristics

For the cross-sectional analysis, a total of 134 participants (64 complete twin pairs), aged 69.5 ± 6.6 years; 58% female, were included. For the longitudinal analyses (4.18 ± 0.34 years follow-up), 88 participants (39 complete twin pairs) were included. Participants were cognitively unimpaired at baseline, with an MMSE of 29 ± 1, and 31 (23%) were classified as Aβ positive on VR (Table 1). Out of all participants, only two participants developed MCI (MMSE of 26) and one participant developed cognitive impairment (MMSE of 23), while all others kept MMSE above 26.^39^

**Table 1.** Cohort characteristics.

	Total cohort for cross-sectional analysis (n = 134)	Longitudinal subset (n = 88)	
	Baseline	Baseline	Follow-up
Age (years)	69.5 ± 6.6	67.3 ± 5.5	71.5 ± 5.9
Sex (females, %)	77 (57.5%)	45 (51.1%)	45 (51.1%)
Complete twin pairs (n)	64	39	39
Visual read positive (%)	31 (23.0%)	17 (19.3%)	34 (38.6%)
Global CBF (mL/100 g/min)	62.4 ± 10.4	62.1 ± 9.5	63.3 ± 9.2
ACA CBF	67.8 ± 10.7	67.5 ± 9.9	69.4 ± 10.0
MCA CBF	66.7 ± 10.4	66.6 ± 9.2	67.6 ± 9.4
PCA CBF	59.7 ± 11.5	59.9 ± 10.2	58.7 ± 9.9
Global Centiloid (mean ± SD)	12.2 ± 20.8	9.1 ± 16.7	10.9 ± 22.1
MMSE (mean ± SD)	29.1 ± 1.0	29.0 ± 0.9	28.9 ± 1.1
Framingham score (mean ± SD)	16.5 ± 3.4	15.7 ± 3.1	n/a
WMH volume (mL)	5.8 ± 7.3	5.7 ± 8.2	9.25 ± 12.01

CBF values after partial volume correction.

### Genetic component of CBF

**Figure 1.**
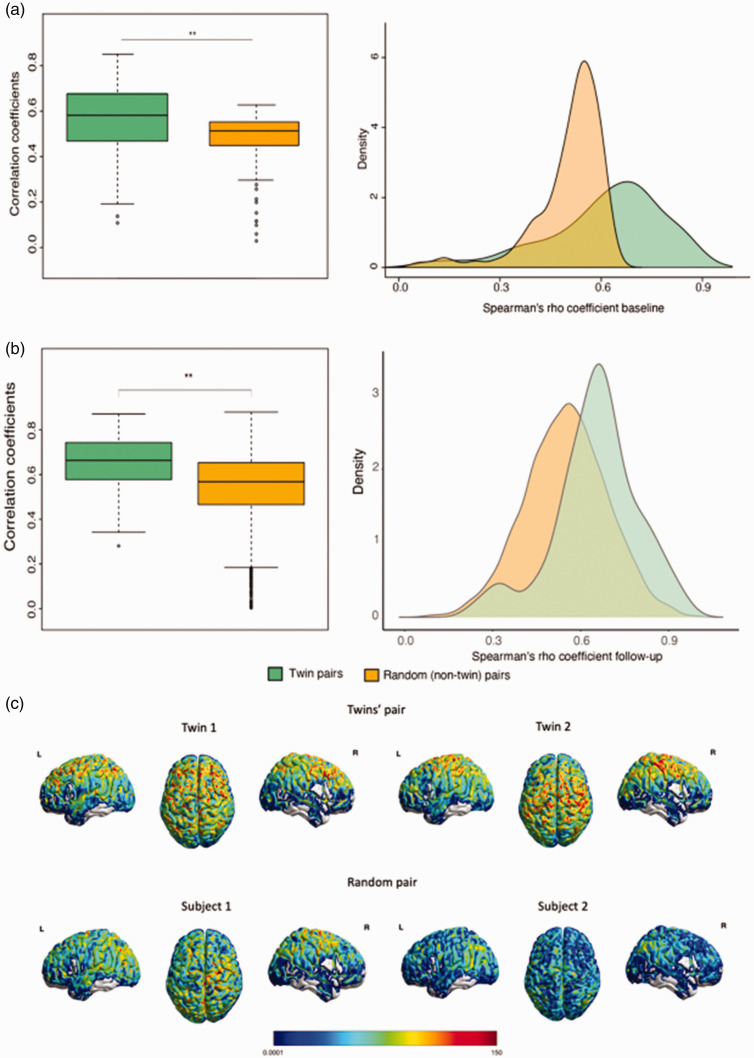
CBF perfusion patterns analysis. The Spearman correlation coefficients of similarity calculated for each twin pair and random pair at both baseline (a) and follow-up (b) (p < 0.01) and (c) Representative CBF images from a pair of twins (top) and a random pair of subjects (bottom).

We first examined within-pair correlations for baseline and delta CBF. For the twin pairs, these were statistically significant for all territories, which was not the case for the random-pairs correlations (Table 2, Supplementary figure 3, Figure 1). When removing outliers that were more than two standard deviations below and above the mean, the correlation did not significantly change (r = 0.48, and r = −0.16 for twin pairs and random pairs respectively).

**Table 2.** The results of the ICC analysis of both baseline and delta CBF, are shown for twin pairs and random pairs.

	Twin pairs		Random pairs	
ICC results	ICC	*p*	ICC	*p*
Baseline
Global GM CBF	0.49	<0.001**	−0.16	0.50
ACA CBF	0.46	<0.001**	−0.03	0.49
MCA CBF	0.47	<0.001**	−0.03	0.49
PCA CBF	0.49	<0.001**	−0.01	0.50
Delta CBF				
Global GM CBF	0.33	0.036*	−0.13	0.653
ACA CBF	0.36	0.030*	−0.21	0.791
MCA CBF	0.36	0.030*	−0.18	0.793
PCA CBF	0.11	0.255	−0.06	0.595

*p < 0.05, **p < 0.001.

Results did not change with using normalized CBF values (*i.e.* vascular territories CBF divided by global CBF). Figure 1 shows CBF perfusion patterns correlations within twin pairs (1a) and random pairs (1b) for both baseline and follow-up. Twin pairs were significantly more similar in CBF perfusion patterns compared to random pairs, both at baseline (t(87) = 3.9, *p < *0.001) and follow-up (t(38) = 5.04, *p < *0.001). Figure 1C presents representative CBF maps of a twin pair and a random pair, respectively. The Spearman correlations of CBF across Hammers regions were, on average, significantly higher for twin pairs than random pairs (p < 0.001), independent of the visit; and at follow-up, were also significantly higher than at baseline (p < 0.001), independent on the group (Supplementary figure 4).

### Vascular and amyloid components of CBF

#### FRS, WMH, and their interaction

No associations were found between all CBF measures and FRS. WMH volumes were negatively correlated with both global and regional CBF at baseline. However, when removing outliers that were more than two standard deviations below and above the mean, the correlation disappeared (Supplementary figure 5). The interaction between FRS and WMH was also not associated with GM CBF (p > 0.05, data not shown). Additionally, we investigated the association between white matter CBF and WMH, which was not statistically significant (p = 0.14, data not shown). When investigating the influence of microbleeds on CBF values, we did not find differences between the three groups (p = 0.784; Supplementary Figure 6), but it is possible to visualize a trend of initial incline and later decline of CBF. When comparing the lacune groups (Supplementary Figure 7), no GM CBF difference was found (p-value = 0.303).

#### Aβ burden and its interaction with FRS

**Figure 2.**
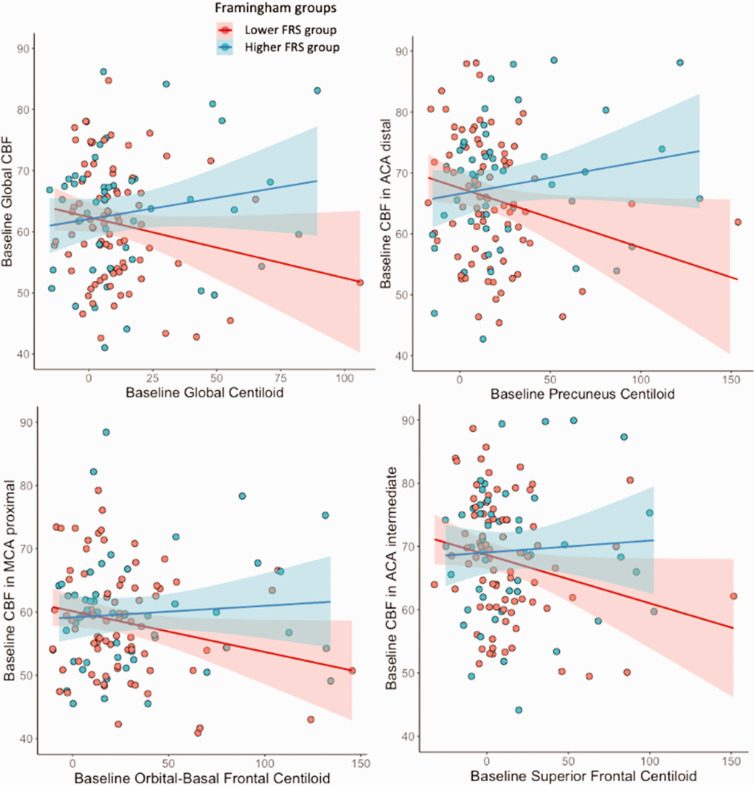
Significant associations between baseline Aβ burden and changes in CBF in the vascular territories, shown with the two Framingham groups. Group 1 (red) = lower Framingham risk scores; group 2 (blue) = higher Framingham risk scores.

Although no direct association was observed between Aβ burden and CBF at baseline, an interaction effect between FRS and Aβ burden on baseline CBF (Table 3) was found. In participants with a high FRS, higher Aβ was associated with increased baseline CBF for all ROIs, except the lingual gyrus (Table 3, Figure 2). However, when excluding outliers that were two standard deviations below and above the mean, the only association that remained statistically significant was between the ACA distal CBF and precuneus amyloid burden (β = 0.046, p = 0.005, Table 3). Longitudinally, baseline Aβ burden in the precuneus was associated with delta CBF in the corresponding vascular territory (β = 0.052, p = 0.017, Supplementary figure 6), but no other Aβ ROI was associated with delta CBF (Supplementary figure 8). However, when removing outliers that were two standard deviations below and above the mean, both precuneus (β = 0.035, p = 0.027) and orbital-frontal (β = 0.003, p = 0.014) amyloid burden were correlated with delta CBF (Supplementary figure 9). Interaction of Aβ with FRS was not associated with CBF changes.

**Table 3.** Cross-sectional CBF GEE results are shown for regional centiloid and WMH volume correlations with CBF.

CBF vs Amyloid burden (GEE analysis)		Amyloid burden		FRS × amyloid burden		FRS × amyloid burden (outliers removed)	
*Predictor (Amyloid ROIs)*	*Dependent (CBF vasc. terr.)*	*β*	*p*	*β*	*p*	*β*	*p*
Global	Global	0.006	0.882	0.013	0.019**	0.010	0.79
Orbital-basal frontal gyrus	MCA proximal	−0.013	0.592	0.020	0.0102*	0.011	0.49
Precuneus	ACA distal	0.162	0.602	0.171	0.0063**	0.046	0.005*
Superior frontal gyrus	ACA intermediate	−0.005	0.863	0.025	0.013*	0.028	0.22
Lingual gyrus	PCA intermediate	0.054	0.562	0.059	0.051	0.028	0.167

ACA: arterial cerebral artery; MCA: middle cerebral artery; PCA: posterior cerebral artery; FRS: Framingham risk scores.

Values were z-scored for normality. x indicates the interaction effect.

*p < 0.05, **p < 0.01.

#### Post-hoc longitudinal CBF analysis by amyloid-status

As a post-hoc longitudinal analysis, we grouped the participants that had both ASL and PET longitudinal data (n = 98) based on their longitudinal visual read Aβ status: stable Aβ negative (n = 77), stable Aβ positive (n = 8), negative-to-positive convertors (n = 13). The stable Aβ-positive participants had the highest delta CBF (Supplementary Figure 10). This group difference was only statistically significant for the ACA distal (the vascular territory corresponding to the precuneus). The stable Aβ+ group had a significantly higher CBF change compared to the Stable Aβ− group (t(8) =−2.8887, p = 0.0189) and to the group that converted to Aβ+ at follow-up (t(14) = −2.3325, p = 0.0341).

#### Influence of APOE4

In the GEE analyses, APOE4 status did not show any influence on the association of CBF with amyloid burden or with WMH volume (p > 0.05, Supplementary Table).

## Discussion

In a genetically informative sample of cognitively unimpaired monozygotic twins, we found: 1) a moderate genetic contribution to brain perfusion and its patterns, 2) an association between WMH volume and baseline CBF, although CBF was not associated with cardiovascular risk scores, and 3) Aβ load was associated with changes in CBF over time, whereas baseline CBF was only associated with Aβ burden through its interaction with cardiovascular risk factors. These findings suggest that even before the onset of cognitive impairment, CBF is already influenced by cerebrovascular, and amyloid proteinopathy components.

The fact that we observed within-pair CBF similarities in both whole vascular territories and spatial distribution patterns suggests a genetic component of both a whole-brain and localized perfusion, respectively. Perfusion patterns correlations were significantly higher within twin pairs compared to random pairs. This was the case both at baseline and follow-up, indicating the robustness of these findings. These results are in agreement with a previous study showing a moderate CBF heritability in a smaller group of 41 monozygotic and 25 dizygotic twins.^19^ Interestingly, CBF patterns were relatively similar even within the non-twin random participants, and this pattern similarity was higher for follow-up than for baseline both within twins and non-twin pairs. These findings suggest age-related perfusion pattern changes, which are in accordance with literature,^40^ meaning that CBF has a common aging pattern, similar to the existence of common brain networks found on functional MRI.^41^ These findings may be interesting to study in more detail in larger population studies.

Our observation that the individuals with the highest WMH volume also had the lowest baseline CBF could fit with several potential mechanisms. SVD could lead to added vascular resistance, impairing CBF, and, on the other hand, hypoperfusion is recognized as a potential cause of WMH.^11^ These findings are encouraging for considering CBF as a possible early cerebrovascular health biomarker, which could help with earlier treatment and avoid irreversible structural damage, e.g. WM lesions.^42^ The disappearance of the correlation between baseline GM CBF and WMH volume after log-transforming the latter might be explained by the fact that this cohort had relatively low WMH volumes and was cognitively healthy. A previous study with AD patients reported an association between WMH and GM CBF^43^ study with similarly aged healthy individuals with hypertension did show an association between WM lesions CBF and WMH volume. However, at this early stage, and because we’re investigating the influence of WMH on GM CBF, we don’t find a substantial effect yet, which might be due to a healthy cognition state and low WMH volume. We did not find an association between WM CBF and WMH volume, which could be attributed to the low WM signal-to-noise ratio of ASL, since the PLD used is relatively short to provide an accurate quantification of WM CBF compared to GM CBF. The absence of a statistically significant effect of the other SVD biomarkers — microbleeds and lacunes — on CBF could be explained by our relatively healthy cohort with relatively low microbleeds and lacunes count.

In contrast with previous studies, we did not observe a direct association between Aβ burden in early accumulation regions and CBF. A possible explanation for this disagreement is that our population was cognitively unimpaired and thus has a relatively small range of Aβ load,^44^ whereas previous studies concerned later AD stages.^13,16,45,46^ However, the interaction of cardiovascular risk (FRS) with Aβ burden was associated with baseline vascular territories’ CBF, suggesting a possible synergistic effect. This could be explained by either inflammation or compensatory mechanisms,^47^ as Aβ accumulation triggers glial activation and the release of inflammatory mediators,^48^ triggering CBF to respond and compensate for Aβ damage. Interestingly, our post-hoc longitudinal group analysis stratified by Aβ visual reading showed that the group of participants with high baseline Aβ burden had a higher increase of CBF than the participants with low baseline Aβ burden. Moreover, the precuneus — one of the earliest AD-related regions for Aβ accumulation and atrophy^46^ — was the region in which Aβ deposition was associated strongest with baseline CBF and CBF changes.^46^ Additionally, when correcting for outliers, both precuneus and orbital frontal amyloid burden were associated with delta CBF regionally, which is in line with the staging cortical amyloid model shown by Collij et al.^17^ These findings are consistent with prior work that found higher regional CBF in participants at risk for AD, but not considered cognitively unimpaired.^49^ This might reflect two possible mechanisms. 1) Increased CBF may reflect an increased demand for nutrients to support increased brain function to compensate for the Aβ accumulation in a preclinical phase of AD;^50,51^ 2) Increased CBF is required to maintain a stable blood-brain barrier (BBB) due to its significant importance in clearing interstitial solutes such as Aβ.^52^

This study has some limitations. Classic twin studies include both monozygotic and dizygotic twins to differentiate between the common genetic and environmental factors that the twins experience. Because we only included monozygotic twin pairs, we could not differentiate genetic effects from the effects of shared environmental factors. A strength but at the same time potential weakness is that we test associations with known dementia biomarkers in a preclinical state. This is a strength as most studies focus on later dementia stages; in which treatment may not be effective anymore. However, this may have limited our findings as the range of pathology — e.g., of Aβ burden — could have been too small for sufficient statistical power to find significant associations. We chose to focus on amyloid burden as a marker of early stages of the disease because we believe it to be more specific than hippocampal atrophy, which can also occur in healthy aging, as a loss of hippocampal volume was seen in clear amyloid-positive subjects.^53^ In order to keep the scope of our study concise, we did not include hippocampal atrophy as an additional biomarker. In addition, we acknowledge that our sample size is relatively small.

Taken together, these findings suggest that, even in a cognitively unimpaired population, CBF variance can be explained partly by genetic, vascular, and amyloid-beta disease factors. These results are encouraging for future studies to investigate the effect of these CBF components on the development of different types of dementias. Furthermore, this work demonstrates the potential value of including CBF in multi-factorial disease trajectory analyses, to investigate their joint impact on cognitive decline.

## Supplemental Material

sj-pdf-1-jcb-10.1177_0271678X231178993 - Supplemental material for Genetic, vascular, and amyloid components of cerebral blood flow in a preclinical population Click here for additional data file. Supplemental material, sj-pdf-1-jcb-10.1177_0271678X231178993 for Genetic, vascular, and amyloid components of cerebral blood flow in a preclinical population by Beatriz E Padrela, Luigi Lorenzini, Lyduine E Collij, David Vállez García, Emma Coomans, Silvia Ingala, Jori Tomassen, Quinten Deckers, Mahnaz Shekari, Eco JC de Geus, Elsmarieke van de Giessen, Mara ten Kate, Pieter Jelle Visser, Frederik Barkhof, Jan Petr, Anouk den Braber and Henk JMM Mutsaerts in Journal of Cerebral Blood Flow & Metabolism
