# Supplementary material for: Genetic, vascular and amyloid components of cerebral blood flow in a preclinical population
Source: J Cereb Blood Flow Metab. 2023 May 26;43(10):1726–36. doi: 10.1177/0271678X231178993 (PMC10581242; doi:10.1177/0271678X231178993)
Supplement: sj-pdf-1-jcb-10.1177_0271678X231178993 - Supplemental material for Genetic, vascular, and amyloid components of cerebral blood flow in a preclinical population [file sj-pdf-1-jcb-10.1177_0271678X231178993.pdf]

Supplementary materials

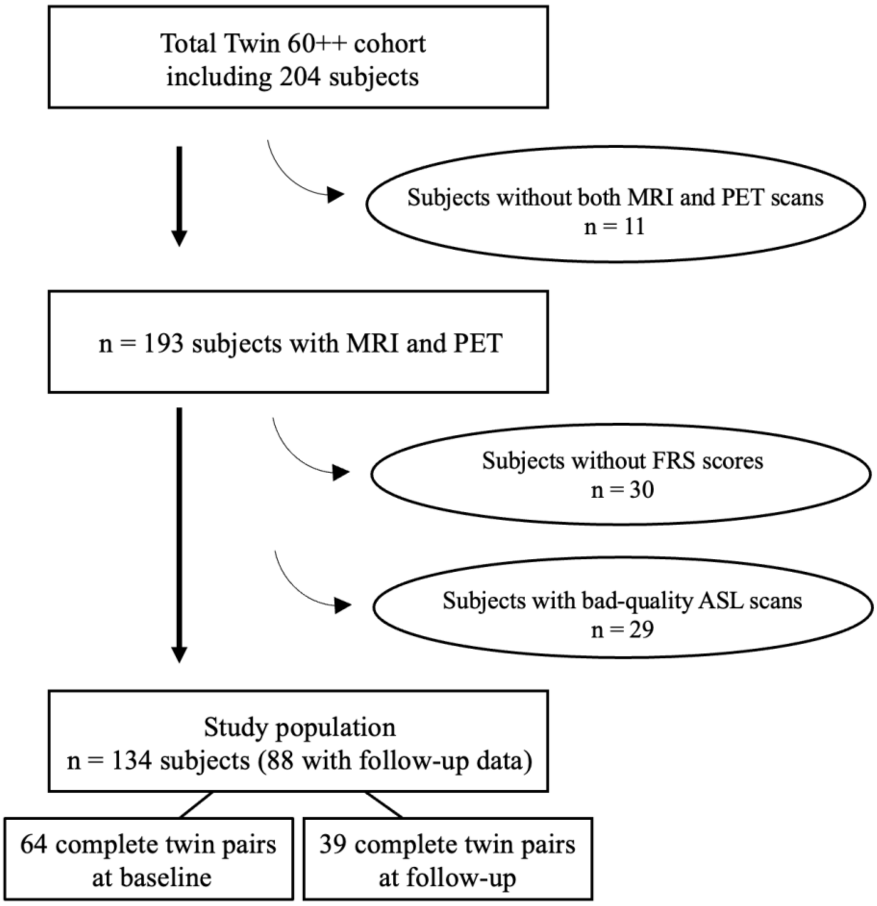

Supplementary figure 1: *Flow diagram of the data inclusion. Follow-up of 4 years. From some twin pairs, if one participant was excluded because of the parameters' absence, that twin was not included in the twin-pair analysis but was included in the population analysis.*

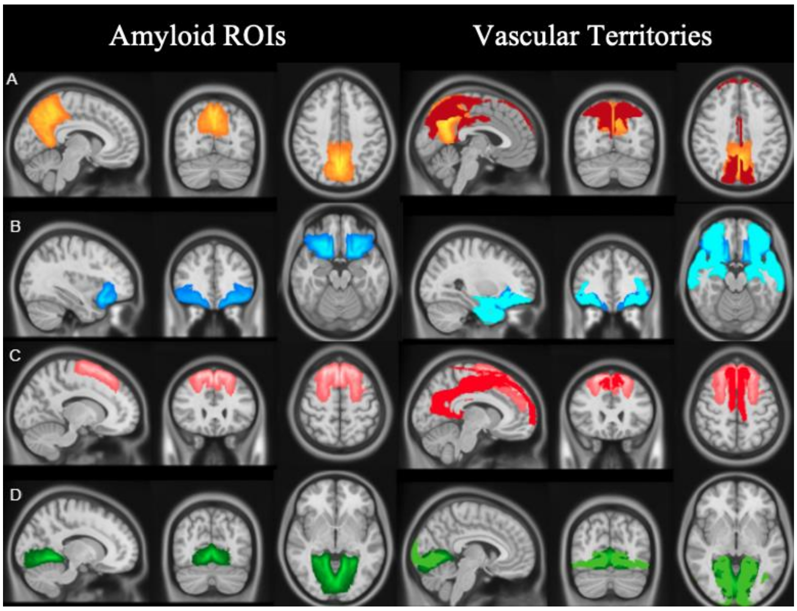

***Supplementary figure 2**: Centiloid regions (left) and anatomical-matching vascular territories (right): A) Precuneus (orange) and anterior cerebral artery (ACA) distal (red); B) Orbital frontal (blue) and middle cerebral artery (MCA) proximal (light blue); C) Superior frontal gyrus (pink) and ACA intermediate (red); D) Lingual gyrus (green) and posterior cerebral artery (PCA) intermediate (light green).*

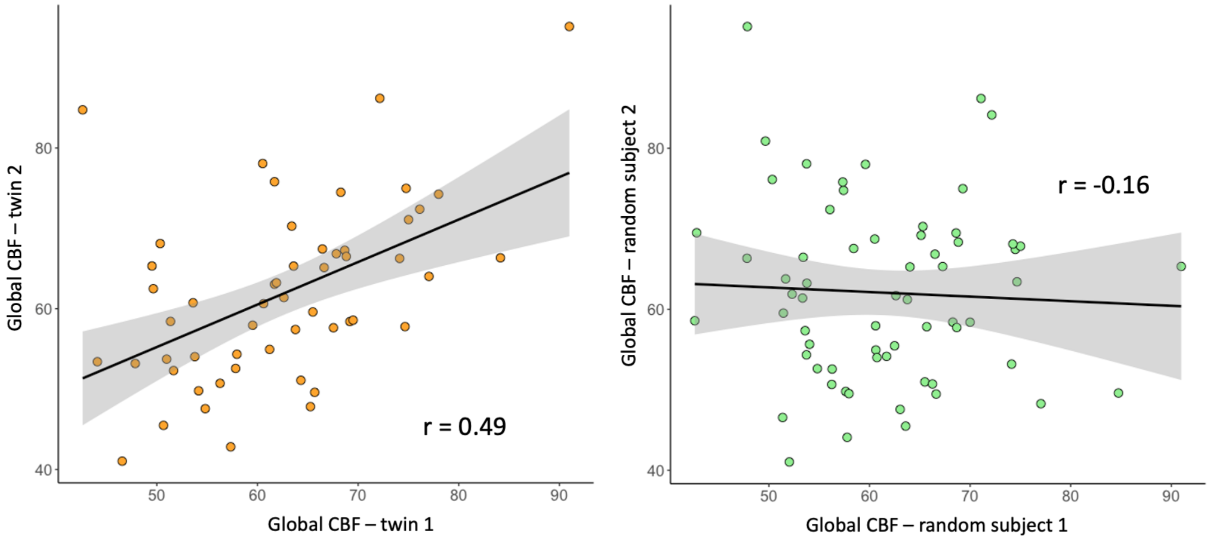

***Supplementary figure 3:** Global CBF values plotted for each twin pair (left; orange) and each random pair in one of the 20 permutations (right; green).*

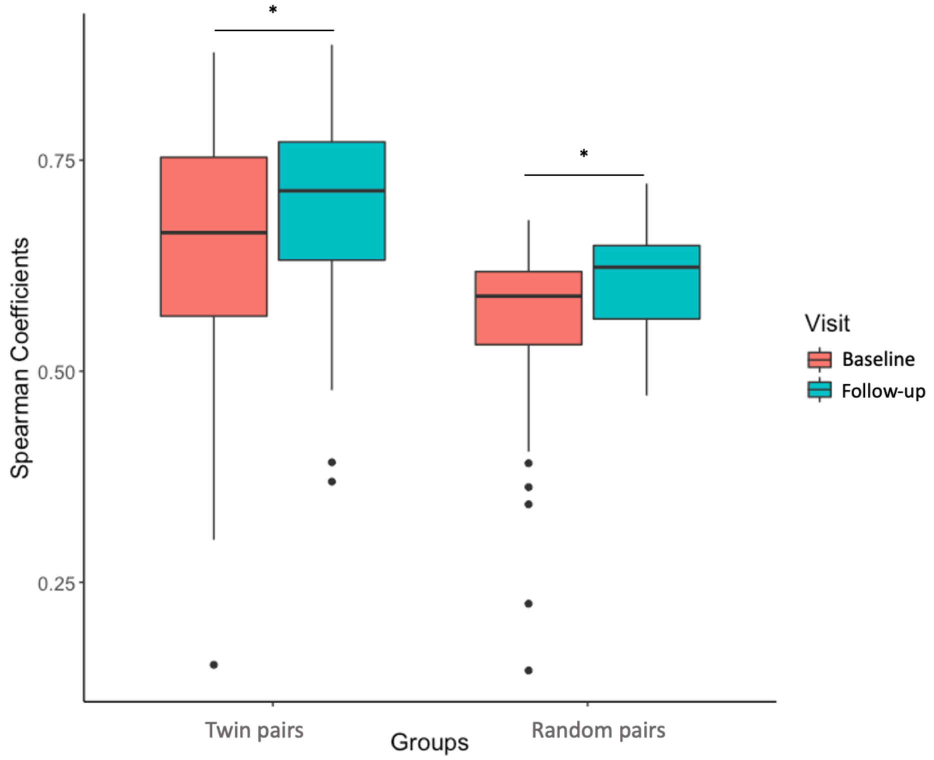

***Supplementary Figure 4**: Analysis of the influence of time and group in the CBF spearman correlations.*

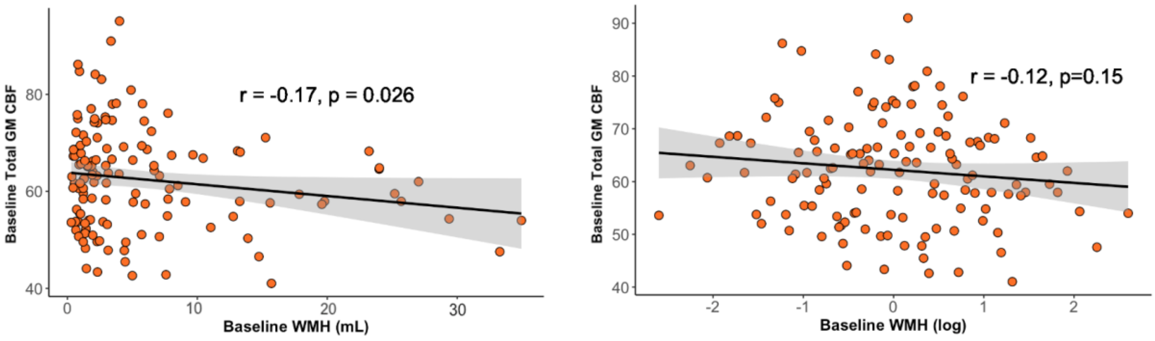

***Supplementary Figure 5:** Global WMH at baseline predicting global CBF at baseline, before (left) and after (right) outlet removal.*

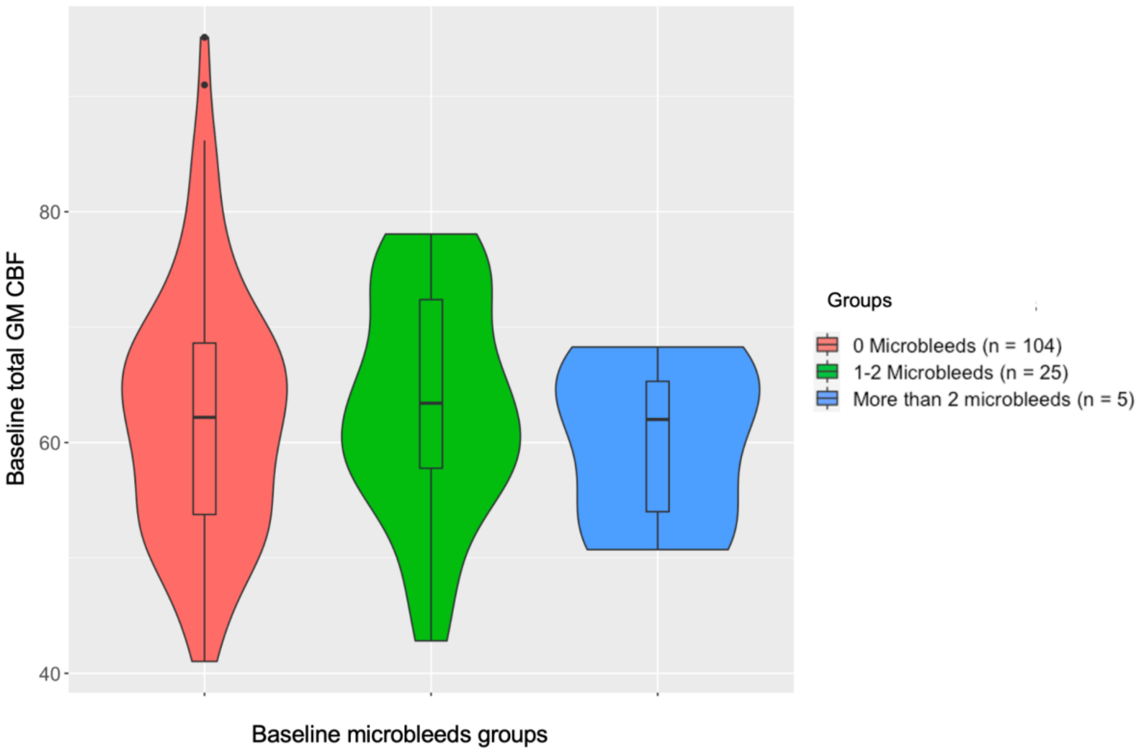

Supplementary Figure 6: Baseline total GM CBF over the three different microbleeds groups.

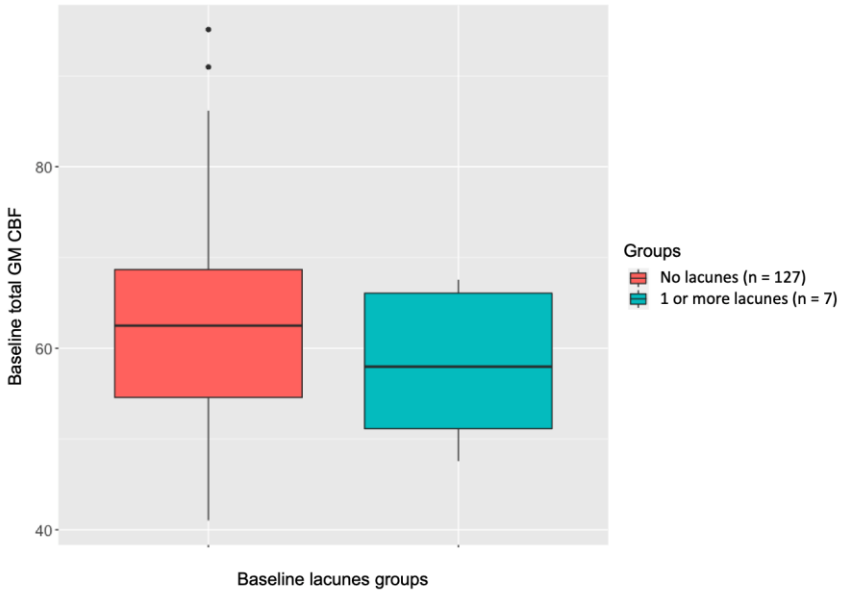

Supplementary Figure 7: Baseline total GM CBF in two different groups without (red) and with (blue) the presence of lacunes.

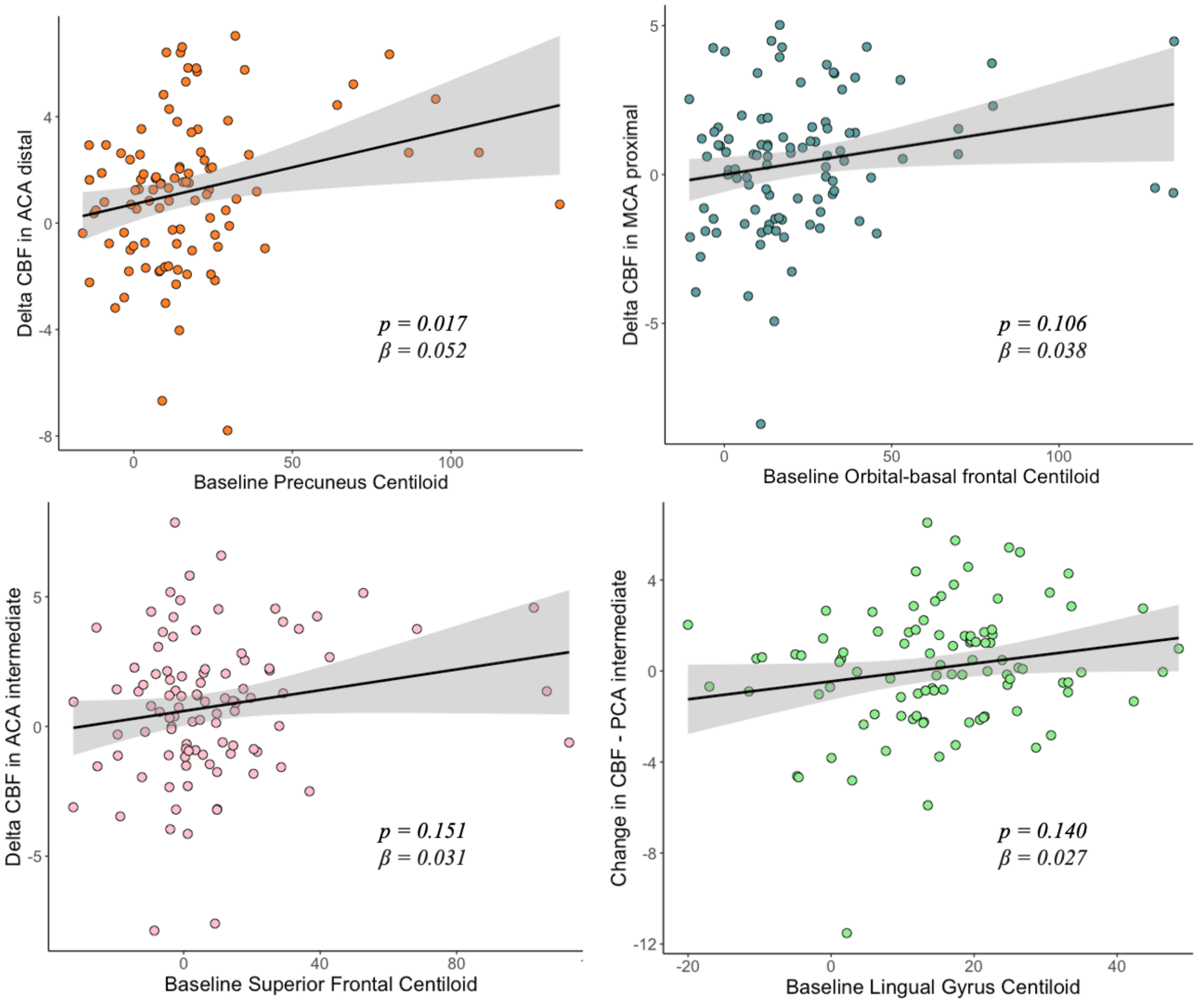

Supplementary Figure 8: *Longitudinal yearly CBF changes in four vascular territories in the four panes (with different colors) predicted by baseline Aβ.*

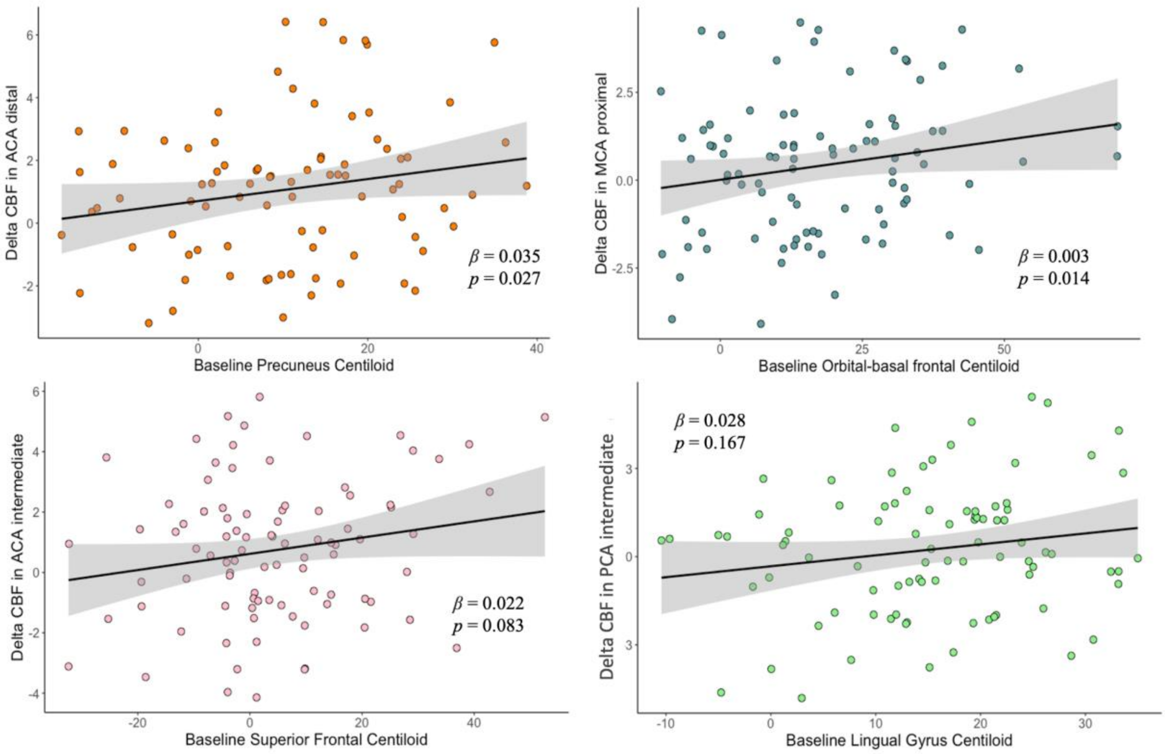

Supplementary Figure 9: *Longitudinal yearly CBF changes in four vascular territories in the four panes (with different colors) predicted by baseline Aβ, after outliers removal*

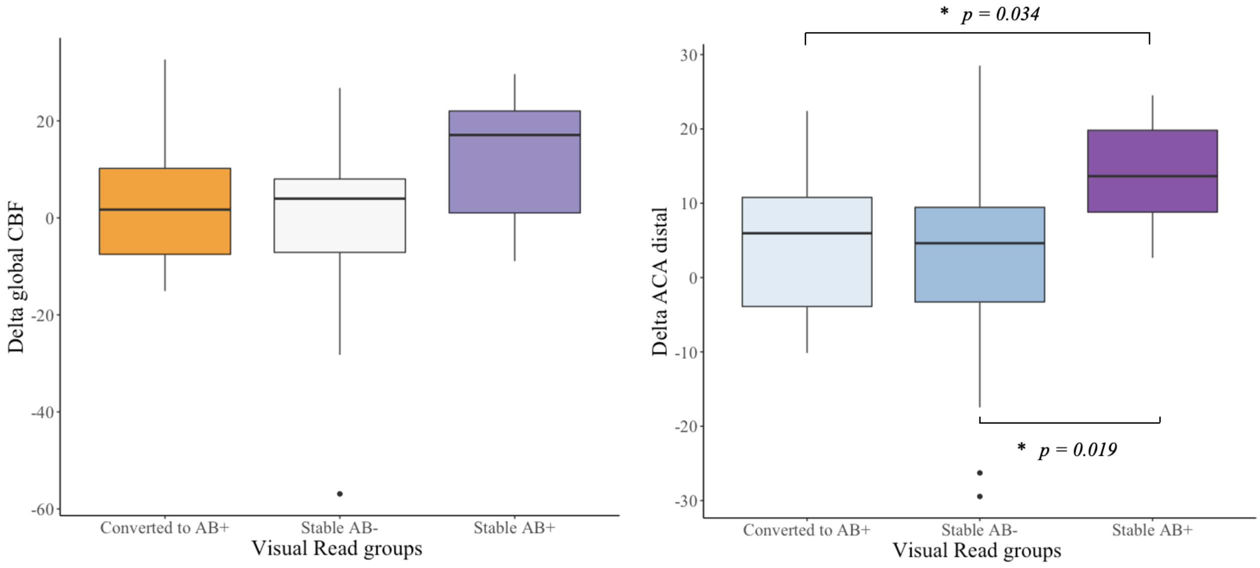

Supplementary Figure 10: *Longitudinal changes in global CBF and arterial cerebral artery (ACA) distal territory shown by the visual reads assessment. Differences between groups are not significant. Stable AB- (n=77), Converters to AB+ (n=15), Stable AB- (n=8).*

	APOE4	Global amyloid burden * APOE4	WMH * APOE4
Total GM CBF	0.167	0.460	0.434
ACA CBF	0.099	0.867	0.355
MCA CBF	0.215	0.882	0.463
PCA CBF	0.303	0.335	0.673

Supplementary Table: *GEE models for investigating APOE4 effects in the associations between CBF and amyloid burden or WMH, for total and vascular territories CBF. The values shown correspond to the p-values of the analyses.*
